# Design, Characterization and Sensitivity Analysis of a Piezoelectric Ceramic/Metal Composite Transducer

**DOI:** 10.3390/mi8090271

**Published:** 2017-09-05

**Authors:** Muhammad bin Mansoor, Sören Köble, Tin Wang Wong, Peter Woias, Frank Goldschmidtböing

**Affiliations:** Laboratory for the Design of Microsystems, Department of Microsystems Engineering (IMTEK), University of Freiburg, 79110 Freiburg, Germany; soeren.koeble@neptun.uni-freiburg.de (S.K.); wth1990@yahoo.com.hk (T.W.W.); woias@imtek.de (P.W.); frank.goldschmidtboeing@imtek.uni-freiburg.de (F.G.)

**Keywords:** piezoelectric transducers, large stroke/high frequency vibrations, nonlinear duffing oscillator

## Abstract

This article presents experimental characterization and numerical simulation techniques used to create large amplitude and high frequency surface waves with the help of a metal/ceramic composite transducer array. Four piezoelectric bimorph transducers are cascaded and operated in a nonlinear regime, creating broad band resonant vibrations. The used metallic plate itself resembles a movable wall which can align perfectly with an airfoil surface. A phase-shifted operation of the actuators results in local displacements that generate a surface wave in the boundary layer for an active turbulence control application. The primary focus of this article is actuator design and a systematic parameter variation experiment which helped optimize its nonlinear dynamics. Finite Element Model (FEM) simulations were performed for different design variants, with a primary focus in particular on the minimization of bending stress seen directly on the piezo elements while achieving the highest possible deflection of the vibrating metallic plate. Large output force and a small yield stress (leading to a relatively small output stoke) are characteristics intrinsic to the stiff piezo-ceramics. Optimized piezo thickness and its spatial distribution on the bending surface resulted in an efficient stress management within the bimorph design. Thus, our proposed resonant transduction array achieved surface vibrations with a maximum peak-to-peak amplitude of 500 μm in a frequency range around 1200 Hz.

## 1. Introduction

Recent progress in the field of aerospace engineering calls for new strategies to be developed aiming to influence the skin-friction drag. Modern research in aviation is focused around the central idea of influencing the aerodynamic flow behavior of the boundary layer near flight-relevant Reynolds number. Possible concepts are the maintenance of laminar flow, delaying the transition from laminar-to-turbulent flow, or influencing the turbulent boundary flow itself. Gad-el-Hak summarized the use of Micro-Electro-Mechanical-Systems (MEMS) actuators for active flow control applications [1]. Kline et al. proposed the most widely recognized idea of a near-wall autonomous and regenerative turbulence cycle, where the formation and interaction of local velocity fluctuations and coherent vortex structures takes place [2,3]. Hutschins et al. argued that at higher Reynolds numbers, the large-scale motion in the outer turbulent boundary layer can have a considerable effect on the near-wall turbulent cycles [4]. The hairpin structures align coherently in groups to form long packets, and packets align coherently to form very large-scale motions (VLSMs). Hence, the main objective of this industry/academia joint project was to research the area of turbulent flow control and identify suitable possibilities to influence the large-scale structures, resulting in reduced frictional drag.

Adrian et al. argued that large amplitude vibrations at frequencies in the region of 1–10 kHz can significantly influence the formation of large-scale coherent structures in the outer turbulent boundary layer [5]. Numerical simulations have also predicted that turbulence can be suppressed by the introduction of a transverse surface wave, leading to shear stress reduction [6]. Laadhari et al. have shown experimentally that turbulence could be suppressed by a spanwise oscillation of a wall section [7]. They produced sinusoidal oscillations (amplitude = 25 mm at frequencies in the region of 2–10 Hz) in a rectangular plate (1 m long, 0.7 m wide, and 10 mm thick) with a crank-shaft system. Roggenkamp et al. utilized an electromagnetic actuation mechanism to create a span-wise transversal surface wave, investigating vibration amplitudes of 250 to 375 μm at a frequency of 81 Hz [8]. Due to high input power density, high working frequency, and extremely high stiffness, piezoelectric actuators find themselves constantly in use for flow control applications. Warsop et al. developed a MEMS-based pulsed jet actuator operated by a piezoelectric cantilever for a flow-separation control experiment [9]. They produced air jets with a maximum speed of 300 m/s at frequencies up to 500 Hz through an orifice, having a diameter of 200 μm, modulated by the operation of a piezoelectric micro valve at 90 V. Haller et al. presented a smart array comprising a thin silicon membrane as a moveable wall with a closed surface for active cancellation of flow instabilities [10]. They utilized uni-morph and cymbal actuators as the excitation source, and fabricated their design with the piezo–polymer-composite technology, creating a surface wave of about 125 μm amplitude having a resonance frequency near 220 Hz. Hence, here we present a metal–ceramic-composite modular array achieving much larger vibrational amplitudes at comparatively higher frequencies. The metallic plate itself becomes the moveable wall in the airfoil surface and creates transverse (either traveling or stationary) surface vibrations within the turbulent boundary layer. The presented design shows system dynamics a factor of 20 faster as compared to what is commonly found in the literature.

## 2. Design Rationale

The actuator design concept entails the desired challenges from the realms of aerodynamics and continuum mechanics. Large amplitude surface vibrations (approximately 1 mm) at high frequencies (approximately 1–10 kHz) are required to align perfectly to an airfoil surface. Moreover, spatial limitations of the airfoil model restrict the maximum height of the transduction array to less than 25 mm. Such high dynamic motion becomes a challenging task once the required input power is considered. Extremely high strokes at high frequencies lead to extremely high accelerations, and therefore huge restoring forces on the moving part of the actuator. Hence choosing a very small dynamic mass—a 150 μm thin brass plate (20×90 mm${}^{2}$, ρ=8440 kg/m${}^{3}$, E= 110 GPa) which would later become a part of the air-foil surface itself—is vital for an efficient actuator design. Surface geometry is designed to be rectangular, which provides an efficient way to cascade the actuators and form a modular transduction array. The actuation surface dimensions are chosen so as to create a traveling wave with a wavelength between 17 and 46 mm, actuating a 120×100 = 12,000 mm${}^{2}$ surface area [11]. In order to ensure smooth alignment and avoid external edges in the boundary layer, the brass plate is glued along all edges with the help of two-component epoxy glue to an aluminum frame.

**Figure 1:**
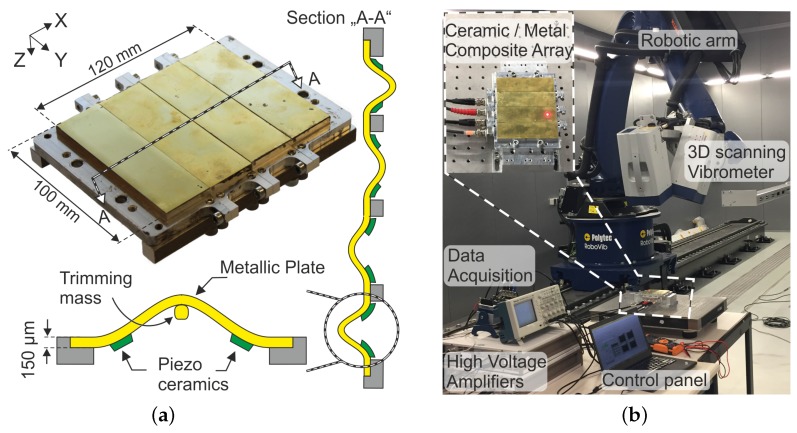
(**a**) 3D and cross-sectional view of the ceramic/metal composite array explaining its actuation principle. (**b**) Measurement setup showing the 3D PSV 500 scanning vibrometer (Polytec GmbH, Waldbronn, Germany) with a robotic arm measuring the entire actuation surface.

The basic actuation principle of the piezoelectric ceramic/metal composite actuator is the “bimorph-effect” (see Figure 1). Essentially, the design consists of two identical piezo elements (the active layer) symmetrically glued to a fully clamped rectangular brass plate (the passive layer). Application of a large external electric field to the piezo elements elongates (or contracts) the active layer while the passive layer tries to retain its original dimension, hence creating an inhomogeneous stress distribution in the ceramic/metal interface. This inhomogeneous stress distribution—resultant of the inherent piezoelectric electro-mechanical coupling—creates a bending moment, leading to an out-of-plane deflection of the plate.

The phenomenon of resonance offers an energy-efficient operational window where energy required for the acceleration is already stored as the elastic potential energy in the system. Under steady-state conditions, only the damping forces need to be overcome by the exciting piezoelectric coupling force. Hence, the piezo elements are excited near the resonance frequency of the brass plate in order to vibrate the bimorph assembly into resonance. A complete analysis on the choice of actuation principle was also published elsewhere [12,13]. As the center displacement of the plate becomes larger than its thickness, in-plane stresses lead to a non-linear restoring force. This geometrically-induced stretching of the mid-plane—commonly modeled as an additional nonlinear spring—allows the actuator to achieve relatively higher strokes in a much broader range of frequencies compared to a linear oscillator with comparable quality factor.

## 3. Finite Element Modeling

Timoshenko has proposed mathematical expressions that can be utilized as “test functions” to analytically calculate the bending surface of a fully clamped rectangular plate [14]. However, symmetric application of the piezo elements to the clamped rectangular plate renders the task non-trivial. Now, a finite set of test functions are required both in *x* and *y* directions to accurately predict the bending surface while estimating the Ritz energy functions (similar to the derivations carried out for piezoelectric compound structures [15,16]).

**Figure 2:**
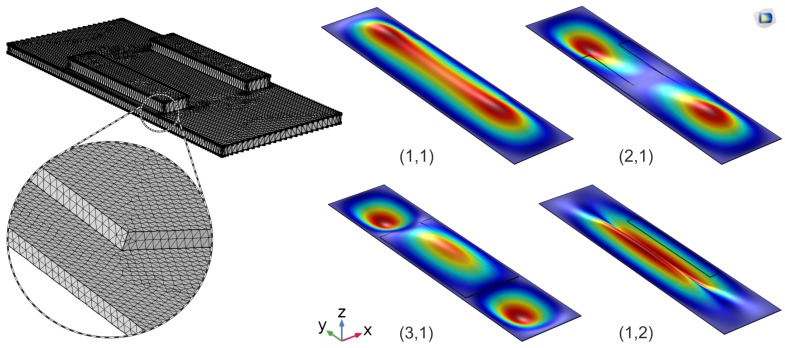
COMSOL Multiphysics implementation of the design with a tetrahedral element mesh and the expected vibration modes of the actuators.

Nevertheless, for such complicated geometries, finite element modeling (FEM) provides the most efficient solution. 3D numerical simulation, conducted using COMSOL Multiphysics 5.1 (COMSOL Inc., Stockholm, Sweden), helped to verify the experimentally obtained results. Here, an optimized device geometry together with the material data provided by the manufacturers (see Table 1) was implemented in a fully coupled electro-mechanical MEMS module for piezoelectric devices. The geometry was built using COMSOL’s in-built Computer Aided Design (CAD) kernel, and the mesh was refined for free tetrahedral geometry with a maximum element size of 100 μm and at the contact boundaries with a maximum element size of 50 μm (see Figure 2). A “fixed-constraint” mechanical boundary condition was used in the solid-mechanics module to create an all-clamped rectangular geometry. An “electric-potential” terminal was used in the coupled electrostatics module generating 1 kV/mm electric field on the piezo-strips. Large signal contributions, which are intrinsic to the nonlinear piezoelectric behavior at high electric field strengths, are also externally added to the numerical model. A fully coupled stationary solver was implemented to solve $9.3\times 10^{5}$ number of degrees of freedom at a relative tolerance of 0.001. The used finite element model allows a geometric parameter sweep to evaluate different device geometries, and numerical simulation results together with the experimentally measured values are presented in the next sections.

## 4. Experimental Characterization

A systematic parametric analysis of the actuator’s design has helped to optimize its performance near the desired frequency of operation. The most influential parameters—considering a large output amplitude at relatively higher frequencies—are (i) the thickness of piezo elements ($h_{p}$) in comparison to brass plate which defines the position of the neutral plane on the cross-section and (ii) the spatial distribution of piezo elements on vibrating composite (characterized as “ΔB”—see Figure 6). A detailed analysis is presented in the next sections highlighting the impact of these optimization parameters on the static and dynamic response of the actuator.

### 4.1. Test Setup

**Figure 3:**
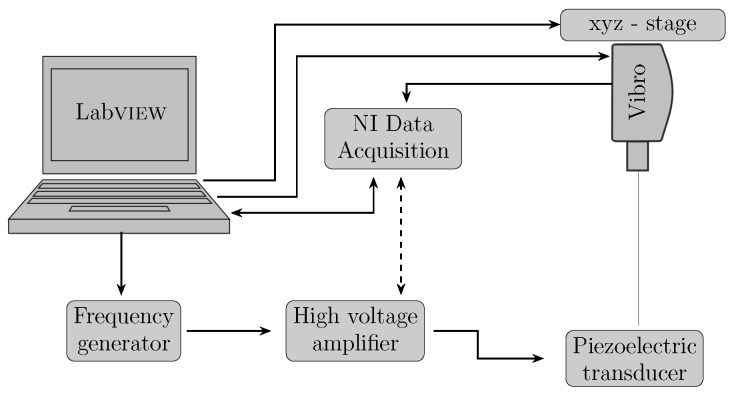
Schematic showing the dynamic experimental measurement setup.

The quasi-static center displacement of the composite actuator up to a maximum of 1 kV/mm applied DC electric field was measured with the help of a 2 mm laser distance sensor (AWL 7/2, Welotech GmbH, Laer, Germany). For dynamic characterization, the piezo elements were excited near the first natural frequency of the brass plate with a sinusoidal signal sweep generated using a function generator. For high electric field actuation, a high-voltage amplifier was used (PZD 300 A, TREK INC., New York, NY, USA). Nonlinear resonant vibrations were measured with a 3D laser vibrometer (PSV 500, Polytec GmbH, Waldbronn, Germany) mounted on top of a robotic arm. The data were recorded and processed in a labVIEW program via a PC-interface (NI-cDAQ 9178, NI9263 and NI9223 (National Instruments, Austin, TX, USA)). The test setup is shown in Figure 3.

### 4.2. Static Response

**Figure 4:**
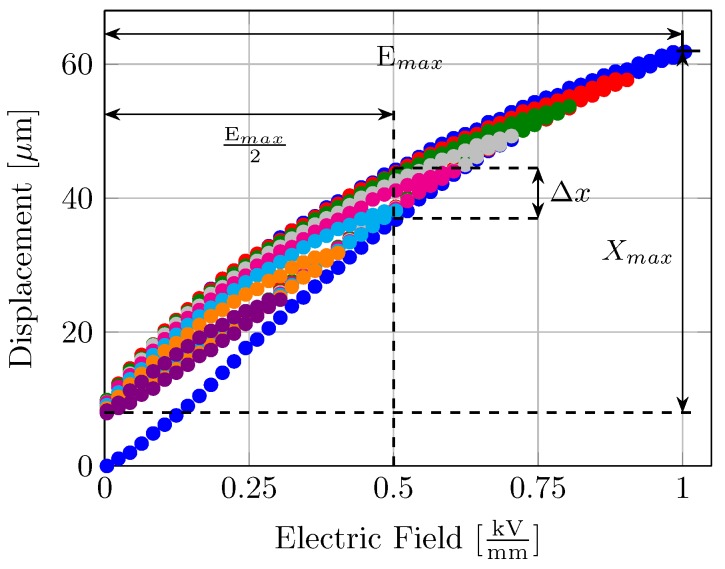
Uni-polar Strain–Electric field curve measured at successively decreasing maximum electric field strengths, showing the intrinsic hysteresis and remnant strain.

Although the actuator is designed to be used in resonance near the natural frequency of the brass plate, static analysis provided vital information which helped to optimize the device geometry. The first quadrant of the so called “butterfly-curve”—associated with intrinsic strain–electric field (S-E) hysteresis in the piezo elements—is shown in Figure 4. The measured center displacement of the plate (corresponding to the lateral strain generated by the piezo elements in the bimorph assembly) follows the so-called virgin curve for an increasing electric field during the first run. The measurement was repeated in a loop with successively decreasing maximum electric field value ($E_{max}$). Depending on the stress history of the transducer, a remnant strain $S_{rem}$ leading to a remnant displacement at zero electric field was observed. Degree of hysteresis is an important figure of merit for piezoelectric transducers, characterizing the reproducibility of output stroke. Uchino [17] defined the degree of hysteresis in a transducer as:

(1) $$Degree\;of\;Hysteresis\;\%=\frac{\Delta x}{X_{max}}\times 100$$

where $X_{max}$ is the displacement at maximum electric field $E_{max}$, and Δx is the difference in displacement for increasing and decreasing paths at half maximum of electric field, $E_{max}/2$. Degree of hysteresis in a piezoelectric transducer relates to the thermal and mechanical losses in the piezoelectric elements and/or the transducer design [18]. The main cause of these hysteretic losses in piezoelectric ceramics is the so-called domain reversal mechanism, which causes internal friction in the ceramic domains [19]. Whereas our proposed transducer design comprises an epoxy glue between the piezo elements and brass plate as well as the plate and the outer aluminum frame, making it the most probable cause of mechanical losses.

The basic goal of the parametric sensitivity analysis was to analyze the static and dynamic actuator characteristics when the active piezo elements were positioned near the clamped edges (ΔB = 1 mm) as compared to the center of the brass plate (ΔB = 6 mm—a common configuration used in many different piezoelectric actuation applications [20,21]). As ΔB increases in steps of 1 mm, the location of the piezo elements moves from the edges towards the center of the plate. The relative position of the piezo elements on the brass plate plays a significant role as we inspect the vibration characteristics of the composite corresponding to the first eigen mode. Six different variations of the parameter ΔB were fabricated (each actuator fabricated twice) and measured under the same experimental conditions.

**Figure 5:**
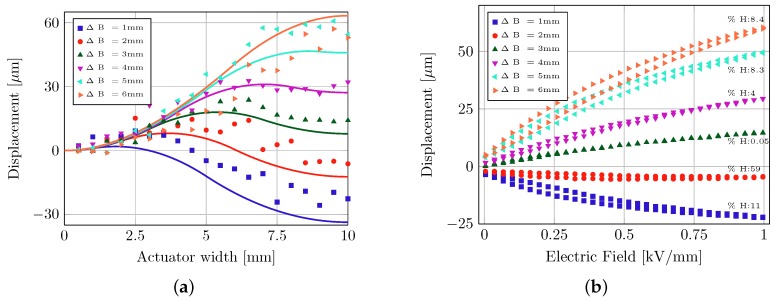
(**a**) Numerically simulated (solid lines) and experimentally measured (bold markers) bending lines of a quarter actuator model. (**b**) Hysteresis curves for a parametric variation of ΔB.

**Figure 6:**
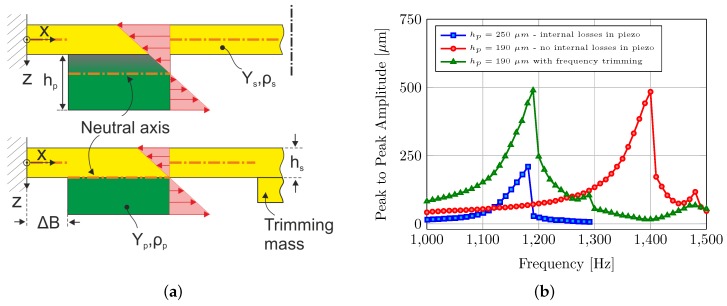
(**a**) Effect of piezo-thickness $h_{p}$ on the non-homogenous strain distribution (solid arrows) and neutral axis position (dotted-dashed line). (**b**) Frequency sweeps near the first resonance frequency of the actuator. All results were measured at an electric field strength of 0.72 kV/mm.

Figure 5 shows numerically simulated bending lines of the quarter representation (shown in Figure 6) which also follow the experimentally measured trend. Relatively large variations of about 15 μm are seen along the ΔB = 1 mm bending curve, which are attributed to a rough actuator surface. Numerically simulated bending lines at the mid-plane also show the characteristic “bimorph-bending” exactly at the position where the piezo-elements are located. Electric field sweeps measured at the mid point of the plate produce the previously mentioned S-E hysteresis curves. Note that these hysteresis curves are always plotted after 10 cycles in order to remove the zero point drift. The remnant strain $S_{rem}$ and hence the remnant displacement also follows the familiar trend based on the relative vibrational amplitude seen directly at the piezo elements. Forward bending of the hysteresis curves is also observed—a common characteristic seen under high electric field operation. Displacement hysteresis among the design variants also show a dependence of the degree of hysteresis on the spatial distribution of piezo elements on the bending surface. Degree of hysteresis—as previously defined—is a characteristic of the width of the hysteresis loop and hence also corresponds to the dissipated energy density per loop [22]. As identical piezo-ceramics are utilized for all six configurations, this change in the dissipated energy density is solely attributed to the placement of piezo elements on the bending surface, and hence their effective strain amplitudes.

### 4.3. Dynamic Response

While the dimensions of the plate itself determine the frequency range of operation, the thickness of the piezo elements decides the location of the neutral axis on the ceramic/metal cross-section (see Figure 6). It is vital to keep the position of the neutral plane exactly at the contact interface between the piezo elements and the brass plate. This helps to avoid the active elements working against their own motion and hence to avoid internal losses. Figure 6 also shows the dynamic characterization results, where the typical hard Duffing-type nonlinearity is clearly observable in the frequency sweeps. Here the thickness of the piezo elements is reduced from 250 to 190 μm, which helps to avoid internal losses in the piezo elements. This change keeps the neutral plane at the contact interface, resulting in more than a factor of two increase in the peak-to-peak output vibrational amplitude. Apart from the piezo elements, there is another thin strip of brass attached exactly at the center of the vibrating plate. This thin strip adds mass to the vibrating assembly without influencing the bending surface, and hence acts a “frequency trimming mass”. This mass helps to compensate the design tolerances and adjust the resonance frequency to the desired frequency of operation.

**Figure 7:**
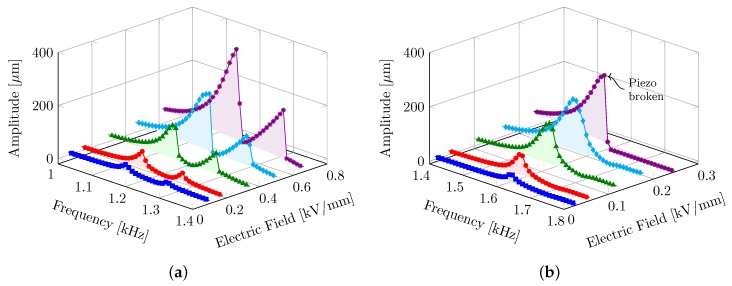
Frequency sweeps with increasing electric field strengths for transducer configurations with piezo elements near the edges (ΔB = 1 mm—(**a**)) and at the center (ΔB = 6 mm—(**b**)) of the plate.

Under quasi-static conditions, the actuation scheme with piezo elements directly placed in the middle of the plate (ΔB = 6 mm) favors the largest absolute bending, as the active elements are farthest away from the mechanically clamped edges (as seen in Figure 5). On the other hand, Figure 7 shows the dynamic response of the transducers under increasing electric field strengths for two extreme configurations (i.e., ΔB = 1 mm compared to ΔB = 6 mm). For high-frequency operations, the piezo elements at the center of the plate tend to break apart quite easily at relatively lower excitation field strengths. Transducers with piezo elements near the edges are relatively softer and have the first resonance vibration mode (1,1) near 1200 Hz. Here we also observe the third resonance peak (3,1), comparatively smaller than the first resonance peak. Depending on the position of the piezo elements, the effective thickness of the bending surface (and hence its stiffness) in comparison to the vibrating mass changes. This leads to an increased resonance frequency for the case ΔB = 6 mm.

## 5. Discussion

**Figure 8:**
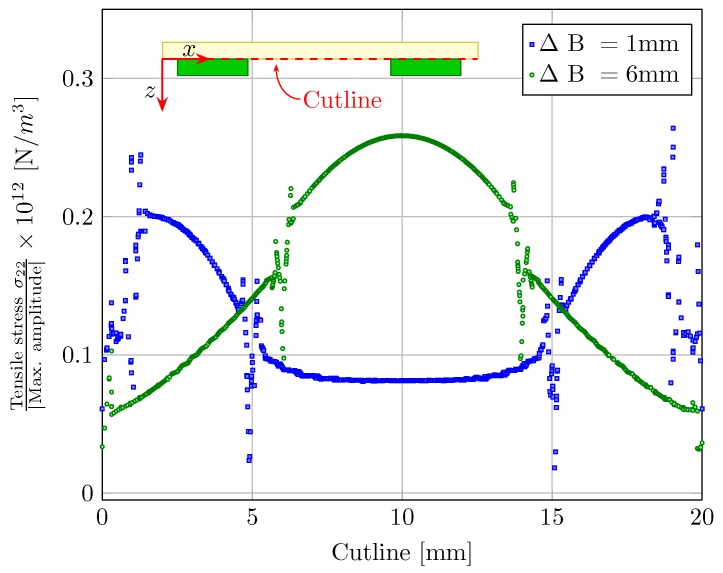
Numerically simulated cost-function (defined in terms of a ratio of tensile stress $\sigma_{22}$ at the piezo to max. absolute output amplitude) across the *x*-axis cutline.

Considering the fact that the first eigen mode of vibration is similar to the static deflection of the transducer, we can draw comprehensive conclusions based on the static FEM simulation results. As piezo ceramics are known to be fragile towards tensile stresses, a “cost function” is defined in terms of the tensile stress $\sigma_{22}$ seen along the active piezo elements and the maximum output absolute deflection of the composite at 1 kV/mm. For large amplitude vibration, minimizing the cost function would result in the strongest actuation with low stress levels in the active piezo elements. Figure 8 shows the numerically simulated cost function under quasi-static conditions for different transducer configurations along the *x*-axis cutline. Note that for clarity the cutline is only shown on a 2D simplification. At the ceramic boundaries along the *x*-axis cutline, a clear jump in the tensile stress distribution is observed. These jumps are accompanied by the irregular spikes, which are presumably a simulation anomaly arising due to a possible stress concentration at the ceramic boundaries (and hence a sudden jump in the neutral axis position). The maximum cost function value along the *x*-axis for ΔB = 6 mm configuration is higher as compared to the ΔB = 1 mm configuration. Here, the piezo elements themselves have to undergo the complete range of vibration as compared to transducers with piezo elements near the edges. Hence, the effective dynamic strain amplitude seen directly at the piezo elements is relatively larger when they are positioned at the middle of the plate. Thus, positioning the piezo elements near the edges allows them to withstand a much higher electric field strength while the composite vibrates in resonance. Due to the breakage of the brittle ceramics under resonant vibrations, positioning the piezo ceramics near the edges of the plate offers a considerable advantage for fast dynamic motion with large vibrational amplitudes.

**Figure 9:**
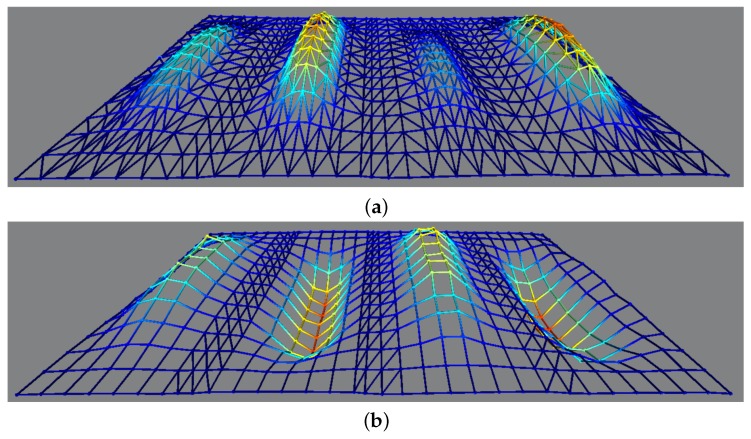
Surface vibrations of the whole array measured with the help of a 3D PSV vibrometer mounted on a robotic arm for in-phase (**a**) and $90^{\circ }$ out-of-phase (**b**) actuation.

Finally, four such transducers are cascaded in a row to form an array capable of creating surface waves. Here the individual actuators are operated with a phase-shifted sinusoidal excitation signal at a frequency of 1200 Hz. Due to the above-mentioned nonlinear hardening behavior, a sweep from lower to higher frequencies is always required in order to vibrate the plate in the higher energy orbit. Figure 9 shows the 3D vibration surface of four such optimized transducers operated with and without a phase shift of $90^{\circ }$ among each other. The vibration surface is measured with the 3D PSV vibrometers mounted on a robotic arm, as shown in Figure 1. The whole actuation surface is divided into a fine rectangular mesh of measurement points and recorded individually. As the phase information is also recorded with the vibrometer, a reconstruction of the measured data with the help of the automated PSV software from Polytec GmbH allows every mode to be visualized individually. Slight differences in the peak-to-peak values are attributed to different resonance frequencies of the transducers, which are minimized by frequency trimming but remain unavoidable.

## 6. Conclusions

Here we have presented a high energy density transduction scheme suitable for extremely fast system dynamics capable of creating either stationary or traveling surface waves in a turbulent boundary layer. Piezoelectric transducers are normally quite stiff and suitable for high-frequency operations. On the other hand, piezo ceramics are known to be particularly sensitive towards tensile loads. For large amplitude vibrations, the limiting factor for maximizing the stroke of the actuator is the mechanical failure of the brittle piezo ceramics, which break away at higher excitation field strengths. As observed in the parametric optimization results, ceramic/metal composite transducer shows the maximum absolute displacement under quasi-static conditions when the active elements are positioned near the middle of the plate. Here, the piezo elements themselves are positioned at the maximum displacement portion of the bending surface. On the contrary, positioning the piezo elements near the clamped edges have the advantage that the piezo-ceramics themselves do not have to undergo the maximum vibrational amplitude when the plate starts to vibrate in resonance. Therefore, the optimization criterion for a fast dynamic application is to have the smallest possible bending stress on the piezo elements while achieving maximum stroke of the vibrating plate.

## Figures and Tables

**Table 1:** Material constants for PZT-5K4 (Morgan Advanced Materials, Ohio, OH, USA) provided by the manufacturer: Morgan Advanced Materials (Ohio, OH, USA).

$\rho \;[\frac{\mathrm{kg}}{m^{3}}]$	ν	Dielectric Constants		Compliance’s $\times 10^{-12}[\frac{m^{2}}{N}]$		Charge Constants $\times 10^{-12}[\frac{m}{V}]$		
8300	0.31	$\epsilon_{33}^{T}$ = 7066	$\epsilon_{11}^{T}$ = 6129	$s_{33}^{E}$ = 20.03	$s_{11}^{E}$ = 15.55	$d_{33}$ = 926	$d_{31}$ = −407	$d_{15}$ = 950
